# A Case Report of Neuroleukemiosis Detected on FDG PET Scan in a Patient of Mixed Phenotype Acute Leukemia Post Bone Marrow Transplantation in Remission

**DOI:** 10.1055/s-0044-1800837

**Published:** 2024-12-05

**Authors:** Ramya Soundararajan, Archana Yadav, Anil Kumar AVS, Hemlata Jangir

**Affiliations:** 1 Department of Nuclear Medicine, Army Hosp Research and Referral, New Delhi, India; 2 Department of Pathology, All India Institute of Medical Sciences, New Delhi, India

**Keywords:** ^18^ F-FDG PET/CT, peripheral nerve, acute leukemia, extramedullary, neuroleukemiosis

## Abstract

Neuroleukemiosis, an exceedingly rare manifestation of leukemia, is defined as peripheral nerve infiltration by leukemic cells. The typical clinical presentation is with peripheral neuropathy and/or chloromatous masses. The diagnosis of this condition is quite challenging, as symptoms usually appear in patients during remission and there are no other signs of relapse. The diagnosis is supported with electrophysiologic testing and imaging and finally established with histopathology and immunophenotyping. We present a case of multineuritis in a patient with mixed phenotype acute leukemia during remission post allogeneic hematopoietic stem cell transplant, where inflamed thickened nerves were detected on positron emission tomography/computed tomography imaging with fluorine-18 fluorodeoxyglucose. A diagnosis of neuroleukemiosis was established via biopsy and immunophenotyping. The literature is reviewed regarding this interesting and rare clinical condition.

## Introduction

Leukemia can infiltrate the leptomeninges, cranial nerves, nerve roots, and very rarely peripheral nerves. This rare infiltration of leukemic cells in the peripheral nerves has been defined as neuroleukemiosis (NLK). 1 The inherent rarity of this disease leads to diagnostic challenges. The literature contains only a handful of case reports of isolated peripheral nerve involvement as first sign of relapse of leukemia without central nervous system (CNS) involvement. We report a rare but classical presentation of NLK in a patient of mixed phenotype acute leukemia (MPAL) in remission following allogeneic hematopoietic stem cell transplantation (ASCT). MPAL is a rare variety of acute leukemia, characterized by biphenotypic or bilineal blasts, that is, the leukemic blasts can express both acute myeloid leukemia and acute lymphoblastic leukemia markers. It accounts for 1 to 3% of acute adult leukemias. 2 3

## Case Report

**Fig. 1:**
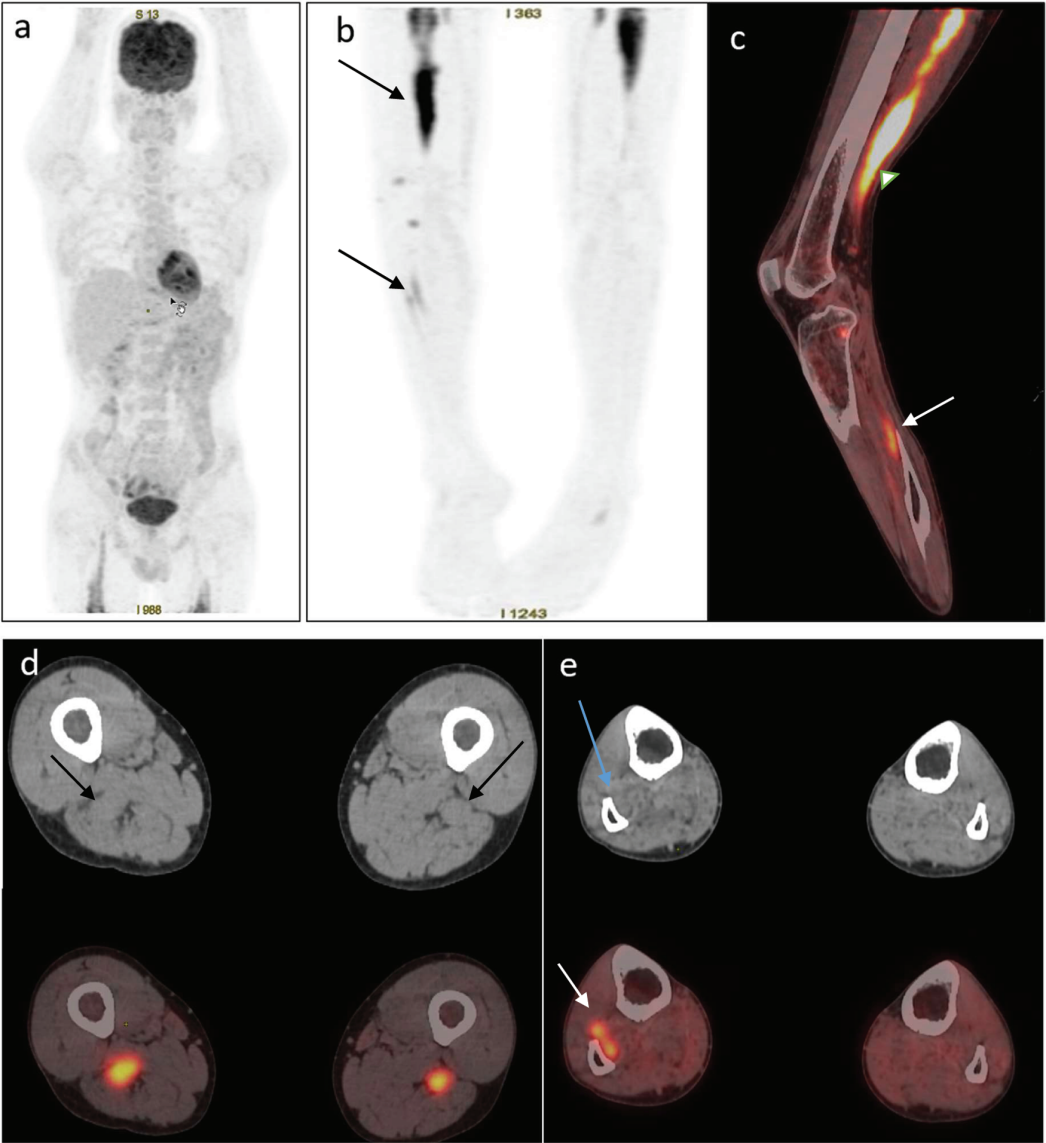
( **a** ) Maximum intensity projection image of the whole body and ( **b** ) lower limbs showing linear increased fluorodeoxyglucose (FDG) uptake in bilateral thigh region (extent in right > left) and right leg region ( *arrow* ). ( **c** ) Fused sagittal positron emission tomography/computed tomography (PET/CT) image of the right lower limb showing diffuse intense FDG uptake in the right sciatic nerve ( *white arrowhead* ) and patchy FDG uptake in the right superficial and deep peroneal nerves ( *white arrow* ). ( **d** ) Axial CT and fused PET/CT image at the upper thigh level showing the bilateral thickened sciatic nerve at the thigh level and increased FDG uptake in the bilateral sciatic nerve. ( **e** ) CT and fused axial PET/CT images at the mid-leg level showing thickened superficial and deep peroneal nerves ( *blue arrow* ) in the right leg with increased FDG uptake.

**Fig. 2:**
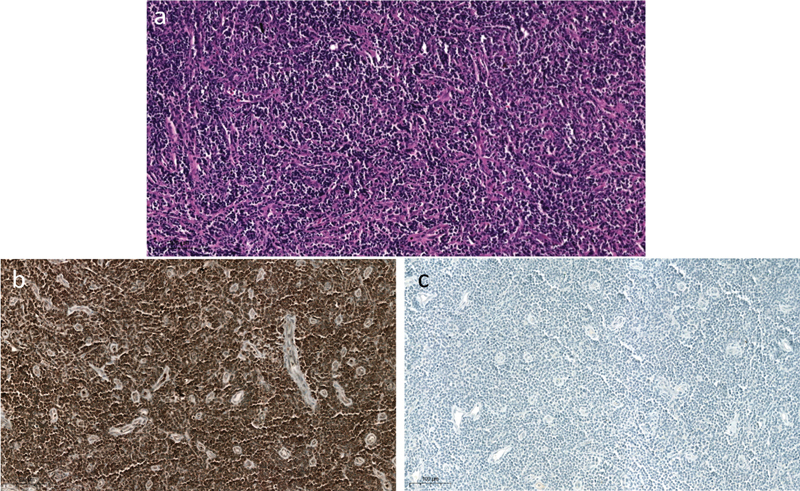
( **a** ) Microscopic images showing diffuse sheets of atypical lymphocytes with varying sizes and shapes, predominant nucleoli, and increased mitotic activity. ( **b** ) Tumor cells show positive staining for CD3. ( **c** ) Tumor cells are negative for CD20.

A 30-year-old man with history of MPAL (myeloid/T lymphoid) treated with chemotherapy and ASCT was in the remission phase for the last 6 months. He developed progressive fatigue, severe pain in both lower limbs, and became wheelchair bound over the next few weeks. On evaluation, he had weakness involving all muscle groups of both lower limbs with a grade III power. He underwent bone marrow biopsy to exclude relapse and color Doppler of the bilateral lower limbs to rule out deep venous thrombosis in view of excruciating pain. Cerebrospinal fluid (CSF) cytology was negative for blasts cells. The patient was referred for a whole-body fluorine-18 fluorodeoxyglucose positron emission tomography/computed tomography ( ^18^ F-FDG PET/CT) scan. The images revealed physiological radiotracer distribution in the brain, liver, kidneys, and urinary bladder with asymmetrically increased linear FDG uptake in the bilateral thigh region and right leg ( Fig. 1 ). The linear uptake in the left lower limb was noted only in the upper thigh region. The increased FDG uptake was along the thickened bilateral sciatic nerves (right > left, maximum thickness of ∼25 mm; maximum standardized uptake value [SUV max] of 13.6 on the right side). The linear uptake in the right lower limb was along the course of the sciatic nerve, extending from the level of ischial tuberosity, coursing through the posterior compartment of the thigh and terminating just above the level of the knee joint. Patchy linear FDG uptake was also noted along the right superficial and deep peroneal nerves ( Fig. 1e ). Apart from FDG uptake in the lower limb nerves, there was no other abnormal tracer uptake. Hence, a provisional diagnosis of NLK was made and the patient was further evaluated. Nerve conduction study of the limbs showed no recordable wave in the bilateral peroneal nerve, suggestive of sensory motor axonal polyneuropathy. Biopsy of the right sciatic, sural nerve, and local muscle was done. Microscopy revealed epineurium showing diffuse sheets of atypical lymphocytes ( Fig. 2a, b ). Muscle biopsy showed group atrophy and was free of tumor. Unfortunately, over the next few days, the patient deteriorated very rapidly and succumbed to the illness after 15 days.

## Discussion

NLK is leukemic infiltration of the peripheral nerves and is an exceedingly rare extramedullary leukemia. It has been described in all types of leukemia but is more common in acute myeloid leukemia, especially monocytic or monoblastic leukemia. 4 In a case series by Reddy et al, five patients of NLK were reported, of which three had acute myeloid leukemia and two had chronic lymphocytic leukemia. 5

Poikayil et al reported a case of concurrent leukemia cutis and NLK as the initial presentation of MPAL, which is extremely rare. 6 Our patient too was a case of MPAL showing positivity for myeloid markers (cluster of differentiation CD117, CD13, and myeloperoxidase) and lymphoid markers (CD2, CD3, and CD7).

NLK usually presents as a peripheral neuropathy and/or a subcutaneous mass/chloroma. 3 In the series by Reddy et al, all patients presented with painful progressive motor and sensory deficits. 5 In our patient, severe pain in the bilateral lower limbs was the predominant feature.

In patients with acute leukemia, extramedullary relapse is rare, occurs much later than bone marrow relapse, and has poor prognosis. It commonly involves the skin, soft tissue, bone, and lymph nodes. 7 Both peripheral nervous system (PNS) and CNS involvements are rare as they have barriers that make the spread of leukemic cells to those sites unlikely. 8 The infiltration of leukemic cells into the CNS occurs directly through CSF and into the PNS through hematogenous spread of leukemic cells across the blood–neural barrier. Isolated NLK without CNS involvement is extremely rare. 9 According to Wang et al, only 10 patients have been reported. 9 In our patient, the CSF study was negative for blast cells. Also, the ^18^ F-FDG PET/CT scan of the patient did not reveal any CNS involvement. The possibility of asymptomatic CNS involvement was ruled out, making it a case of isolated NLK.

NLK commonly presents months or years after remission as a form of disease relapse, as the PNS acts as a “ *reservoir* ” for leukemic cells. Nerve–blood barrier protects the leukemic cells in PNS from immune system and chemotherapy. 4 Although rare, it can also present during the acute phase or even as the first manifestation. 3 5 Voin et al reported two cases of NLK in post bone marrow transplant patients during the remission phase. 8 Our patient was also in the remission phase post ASCT for 6 months.

Differential diagnosis for NLK includes Guillain–Barre syndrome, neurotoxicity from chemotherapy, and abscess. Nerve conduction studies can help differentiate axonal damage from demyelination. 9 In our patient, the nerve conduction study/electromyogram revealed nonrecordable waves in the peripheral nerves, ruling out demyelination.

Another differential diagnosis is graft versus host disease (GVHD). The classical targets of acute GVHD are skin, intestinal tract, and liver. Chronic GVHD may involve additional organs (eye, oral mucosa, lung, fascia, and genital tract) and mimic autoimmune diseases. 10 Biochemical parameters, radiological imaging, upper gastrointestinal endoscopy, and sigmoidoscopy were normal in our case. The patient had no skin lesions or rashes over the body. GVHD was ruled out and NLK was confirmed by biopsy.

Nerve biopsy is the gold standard for the diagnosis of NLK. Owing to the invasiveness of the procedure, it is not recommended if there is concern that the procedure could further deteriorate neurological deficit. 3 In such cases, the diagnosis depends on high index of clinical suspicion supported by imaging modalities such as magnetic resonance imaging (MRI) for chloromas and ^18^ F-FDG PET/CT for demonstrating hypermetabolism in leukemic blasts. Also, patchy involvement of the peripheral nerve and absence of chloromas can result in false-negative biopsy results. 11

Glucose transporter type 3 (GLUT-3) is the major glucose transporter on the neuronal surface and increased FDG uptake in peripheral nerves can be observed in various conditions, more commonly due to neurolymphomatosis, NLK, and malignant peripheral nerve sheath tumors. Uncommon benign conditions include schwannomas, chemotherapy-induced polyneuropathy especially vincristine, inflammatory process due to complex regional pain syndrome, multiple neuritis due to infection, and chronic sciatica. 12 13 14 15 16 17 18 All of these conditions have to be ruled out based on history, clinical presentation, radiological findings, nerve conduction studies, and biopsy.

^18^ F-FDG-PET/CT is a useful, noninvasive tool to detect extramedullary sites of leukemia. 10 Hence, a PET/CT prior to nerve biopsy should be contemplated to look for most metabolically active site and avoid false negatives. Esteller Gauxax et al demonstrated hypermetabolism in sciatic, facial, and right median nerve in a case of acute myeloid leukemia during remission. 4 Kiyoki et al also demonstrated accumulation of ^18^ F-FDG in multiple peripheral nerves in a patient with acute myeloid leukemia during induction therapy. 19 In our patient, there was diffuse hypermetabolism in the right sciatic nerve. However, there was patchy involvement of the right peroneal nerve, and, hence, the site of biopsy was decided based on the PET/CT findings.

## Conclusion

In conclusion, symptoms suggestive of peripheral neuropathy in leukemia patients should be considered as a “red flag” even in the remission phase. In the existing literature, only a few cases of MPAL, in remission, presenting with NLK have been reported. Our case highlights the importance of ^18^ F-FDG PET/CT scan as an excellent imaging modality in NLK as it can support the diagnosis of extramedullary manifestation of leukemia. It can guide for the nerve biopsy site accurately and prevent the chances of false negatives.
